# Analysis of *MYO1H* Gene Polymorphism in Skeletal Class-III Malocclusion Due to Mandibular Prognathism

**DOI:** 10.1055/s-0041-1731066

**Published:** 2021-06-25

**Authors:** Anjana Atteeri, Praveen Kumar Neela, Pavan Kumar Mamillapalli, Vasu M. Sesham, Sreekanth Keesara, Jaya Chandra, Udayini Monica, Vasavi Mohan, Shubhangi Miryala, Fatema A. Khan, Priyanka Makthal

**Affiliations:** 1 Department of Orthodontics, Kamineni Institute of Dental Sciences, Narketpally, Telangana, India; 2 Department of Genetics and Molecular Medicine, Vasavi Medical and Research Centre, Hyderabad, Telangana, India

**Keywords:** mandibular prognathism, gene, *MYO1H*

## Abstract

**Background** Mandibular prognathism (MP) is a craniofacial deformity resulting from the combined effects of environmental and genetic factors. Although various linkage and genome-wide association studies for mandibular prognathism have identified multiple strongly associated regions and genes, the causal genes and variants responsible for the deformity remained ambiguous.

**Aim** This research work was aimed to study the association between polymorphism rs10850110 of the *MYO1H* gene and skeletal class-III malocclusion in our local population.

**Materials and Methods** Thirty patients with skeletal class III due to mandibular prognathism in the study group and 30 patients with skeletal class I in the control group were selected for this study. These patients were from both sexes and above age 10 years. Based on the cephalometric values, patients were categorized into study and control groups. SNB (angle between sella, nasion and point B at nasion) greater than 82 degrees with an ANB (angle between point A, nasion and point B at nasion) of less than 0 degrees in the study group and ANB (angle between point A, nasion and point B at nasion) of 2 to 4 degrees in the control group were categorized. The polymorphism (rs10850110) of the *MYO1H* gene was genotyped using polymerase chain reaction and restriction fragment length polymorphism. Associations were tested with SNP exact test using SNPstats software.

**Results** The single-nucleotide polymorphism rs10850110 showed a statistically significant association with mandibular prognathism. The G allele of marker rs10850110 (5′ of myosin1H *- MYO1H* ) was overrepresented when compared with the “A” allele in mandibular prognathism cases ( *p* < 0.0001), and this was very significant.

**Conclusion** These results suggest that the rs10850110 polymorphism of the *MYO1H* gene is associated with an increased risk for mandibular prognathism.

## Introduction

Malocclusion can either be skeletal or dental with disproportions between the size and shape of the jaws or between the size and number of teeth which would produce crowding or spacing. The skeletal class-III malocclusion phenotype is heterogeneous and is usually characterized by maxillary retrusion (midface retrusion), mandibular protrusion (mandibular prognathism), or a combination of these and can occur as an isolated trait or as a part of a syndrome. 1 Individual presenting with class-III malocclusion due to an underlying skeletal discrepancy (maxillary deficiency and/or mandibular prognathism) experience more challenging problems that result in decreased physical, social and psychological health. The phenotype may be noticeable at an early age and generally becomes progressively more evident with growth. The prevalence of mandibular prognathism varies with age, sex, and ethnicity with the highest prevalence in individuals of the East Asian population (15–23%), moderate in Africans (3–8%), and lowest in Caucasians (0.48%- 4%) and gender with a greater female predilection. 2 3

In orthodontics, one of the most challenging aspects of treating patients is predicting mandibular growth, especially in patients who show more pronounced characteristics of mandibular development. The etiology of mandibular prognathism is not understood completely, but it is well known that both genetic components and environmental factors contribute to its development. In some previous studies conducted on family members and twin siblings, it is well documented that there is a strong link between mandibular prognathism and genetics, accounting mainly for the polygenic model of inheritance. 4

Since genetic factors could contribute to the etiology of skeletal class-III malocclusion, the identification of predisposing gene variants would help in predicting the condition and helps in early prevention or intervention. Although various genetic linkage analysis and genome-wide association studies have identified many genes and loci associated with mandibular prognathism, the genes underlying the risk of mandibular prognathism in the general population remain ambiguous, leaving some impetus to search for new candidate genes.

Single nucleotide polymorphisms (SNPs) in specific genes have been associated with the incidence of various skeletal malocclusions. SNPs are single base-pair changes in the DNA sequence that occur with high frequency in the human genome. Thus, it is typically used as markers of a genomic region in genetic studies. 5 Association studies found positive correlations for mandibular prognathism and genes *EPB41, SSX21, MYO1H, COL2A1, TGFB3, PLXNA* , and *LTBP2* within the locus 12q13-q24. 6 Previous studies on individuals at different geographical locations demonstrated an association between a marker rs10850110 in *MYO1H* at locus 12q24.11 and the skeletal class-III phenotype. However, due to differences in genetic backgrounds, the genetic association found in one population may not apply to other populations. Hence, there is a need to do replication studies to determine the genotype and allele distribution in different populations which thereby provides data that could be used as a foundation for future research. Hence, the present study was aimed to determine the role of SNP rs10850110 located on the *MYO1H* gene in skeletal class-III malocclusion in our population.

## Materials and Methods

Male and female patients with age above 10 years, undergoing treatment in the Department of Orthodontics and Dentofacial Orthopaedics, Kamineni Institue of Dental Sciences, Narketpally, Telangana, India., were chosen for the study. The study was approved by the Institutional Ethical Committee (no.: KIDS/IEC/2018/20) and by the Institutional Research Board. Informed consent was obtained from all patients or parents/legal guardians, in the case of minors, before they entered this study. Each patient's clinical aspects and pretreatment lateral cephalometric X-rays were assessed for eligibility. Sixty patients, 30 each in the study group (13 females and 17 males) and the control group (23 females and 7 males), were selected based on the inclusion and exclusion criteria. The inclusion criteria for the study group were cephalometric measurements of angle SNB (angle between sella, nasion and point B at nasion) greater than 82 degrees with an ANB (angle between point A, nasion and point B at nasion) angle (point A-nasion-point B) of less than 0 degrees. The inclusion criteria for the control group were skeletal class-I patients with ANB (angle between point A, nasion and point B at nasion) angle between 2 and 4 degrees. Exclusion criteria for both groups comprised of the presence of any growth disturbances, syndromes, cleft lip and palate, missing teeth, poor quality of radiographic records, consent form not signed, and trauma. Borderline skeletal class-III patients with an ANB (angle between point A, nasion and point B at nasion) of 0 to 2 degrees were also excluded from the study.

Venous blood of 3 mL was collected from cubital fossa of all the patients in an Ethylenediaminetetraacetic acid (EDTA) vacutainer, and these vacutainers were transported in ice packs to maintain a temperature of 2 to 8°C till it is reached the genetic laboratory. DNA isolation, amplification and genotyping were done for all the sixty samples at Department of Genetics and Molecular Medicine, Vasavi Medical and Research Centre, Hyderabad, Telangana, India, under the guidance of a geneticist. DNA isolation/extraction was performed by salting out method 7 and DNA extract was checked to know the quality of genomic DNA using 0.8% agarose gel electrophoresis. This DNA product was then subjected to amplification by polymerase chain reaction (PCR) using the TaqMan master mix (24 µL) containing water (17.75 µL), buffer (2.5 µL), deoxynucleotide triphosphates (dNTPs) (0.5 µL), MgCl _2_ (2 µL), forward primer (0.5 µL), reverse primer (0.5 µL), Taq polymerase (0.25 µL), and to it, 1 µL of DNA template was added. The gene fragment (291 bp) of *MYO1H* containing the rs10850110 SNP (12q24.11) marker was amplified using the primers, a forward primer 5′ACTTTGCCTTCCCCTGGTTA3′, and a reverse primer 5′CTGAGGCAGGAGGATTGTCT3′. The standard amplification conditions were initial denaturation at 95°C for 5 minutes, followed by 35 cycles of 95°C for 45 seconds (denaturation), 60°C for 30 seconds (annealing), and 72°C for 1 minute (extension), with the final extension of 72°C for 10 minutes. The PCR reaction protocol and the primer sequences were adapted from the previous study. The PCR product was then electrophoresed using 2% agarose gel electrophoresis and was observed under ultraviolet (UV) light using the gel documentation system. The amplified PCR product is then subjected to restriction fragment length polymorphism (RFLP) using polyacrylamide gel electrophoresis (PAGE) to detect the genotypes.

### Statistical Analysis

Using SNPStats software 8 (version 1.40.0), which is a simple and ready to use software designed to analyze genetic epidemiology studies of association using SNPs, the distribution of genotype and allele with SNP allele frequencies, SNP genotype frequencies, and SNP exact test for Hardy–Weinberg equilibrium in cases and controls was statistically analyzed. Odds ratios (ORs) and 95% confidence intervals (95% CIs) were calculated using wildtype genotypes or alleles as reference groups. A *p* -value of less than 0.05 was considered statistically significant ( *p* < 0.05).

## Results

**Fig. 1:**
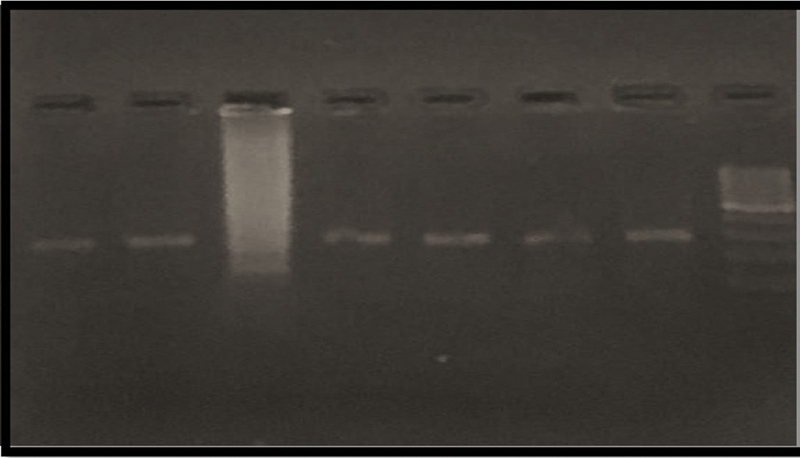
Amplified PCR products visualized using 2% agarose gel electrophoresis. PCR, polymerase chain reaction.

**Fig. 2:**
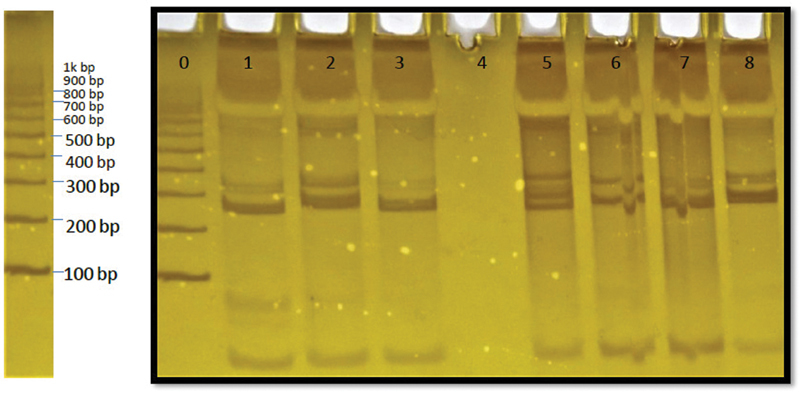
Gel template showing different genotypes. 100 bp (base pair) DNA ladder is shown on the left-hand side. Lane 0: ladder, lane 1, 3, 6, 7: GG genotype, lane 2, 8: AA genotype, lane 5: GA genotype.

A total of 60 patients, 30 each in the study and control group, were evaluated. Fig. 1 shows the PCR products of *MYO1H* on 2% agarose gel for the detection of the rs10850110 polymorphism. The PCR products had acceptable quality and quantity and were selected for genotyping by restriction fragment length polymorphism using polyacrylamide gel electrophoresis. Fig. 2 represents the gel template with different genotypes. The homozygous or heterozygous polymorphic allele was assessed by observing the band position and relating it with the ladder of 100 bp (base pair) in the gel template. The homozygous GG genotype is presented as a single band at 263 bp, while the heterozygous AG genotype is presented as a double band at 263 and 291bp. However, the homozygous AA genotype observed was relatively rare and presented as a single band at 291 bp. The band above 300 bp was not considered since it is a missense mutation.

Tables 1^2^,3 shows the distribution of genotype and allele with SNP allele and genotype frequencies, SNP exact test for Hardy–Weinberg equilibrium and SNP association with response status in both cases and controls respectively. The ancestral allele G was overrepresented in comparison to the A allele in both the groups, and this was significant. The GG genotype was found to be significantly higher than the AA and GA genotypes in both cases and controls. The difference in the frequency of this genotype between the groups (cases and controls) was not significant. The genotypes in the overdominant model also show an association with mandibular prognathism with an OR at a 95% CI was 1.00 with a *p* -value of 0.72.

**Table 1:** SNP allele and genotype frequencies

Genotype and allele	All patients		Status—cases		Status—controls	
	Count	Proportion	Count	Proportion	Count	Proportion
Allele						
G	75	0.62	35	0.58	40	0.67
A	45	0.38	25	0.42	20	0.33
Genotype						
A/A	20	0.33	11	0.37	9	0.3
G/A	5	0.08	3	0.1	2	0.07
G/G	35	0.58	16	0.53	19	0.63

Abbreviations: A, adenine; G, guanine; SNP, single-nucleotide polymorphism.

**Table 2:** SNP exact test for Hardy–Weinberg equilibrium ( *n* = 60)

Genotype and allele	GG	GA	AA	G	A	*p* -Value
All patients	35	5	20	75	45	< 0.0001
Status = cases	16	3	11	35	25	< 0.0001
Status = controls	19	2	9	40	20	< 0.0001

Abbreviations: A, Adenine; G, Guanine; SNP, single-nucleotide polymorphism.

**Table 3:** SNP association with response status ( *n* = 60)

Model	Genotype	Study group (%)	Control group (%)	Odds ratio (95% CI)	*p* -Value
Codominant	G/G	16 (53.3)	19 (63.3)	1.00	0.72
	G/A	3 (10)	2 (6.7)	1.78 (0.26–12.01)	
	A/A	11 (36.7)	9 (30)	1.45 (0.48–4.38)	

Abbreviations: A, adenine; CI, confidence interval; G, guanine; OR, odds ratio; SNP, single-nucleotide polymorphism.

The G allele of marker rs10850110 (5′ of myosin1H *—MYO1H* ) was overrepresented when compared with the “A” allele in mandibular prognathism cases ( *p* < 0.05), and this was very significant. However, the difference in frequency between the groups was not very significant. Also, the A allele of marker rs10850110 was higher in proportion in cases when compared with controls.

## Discussion

Mandibular prognathism is a common craniofacial deformity characterized by either mandibular protrusion or maxillary retrusion or a combination of both. It is mainly due to the overgrowth of the mandible in the sagittal direction. 9 Mandibular growth is regarded as a result of the combined effect of environmental and genetically predetermined intrinsic factors. Research on growth and development has shown that heredity and the mechanical modulation of growth and development share a common pathway via genes. 10 Singh, in his review article, stated that comorphologies of craniomaxillary and mandibular complexes are likely dependent on candidate genes that undergo gene-environmental interactions to bring about mandibular prognathism. 1 Thus, mandibular prognathism is multifactorial and has a complex trait. Since there is consensus that the development of mandibular prognathism is genetically predetermined, various researchers (Wolf et al and EI-Gheriani et al) have investigated the candidate genes governing mandibular development. 11 12 Various genome-wide linkage and genome-wide association studies conducted, the results of which provide evidence that many genomic areas may harbor a large number of genes suggested contributing to class-III malocclusion. Genome-wide linkage and association studies also found positive correlations for mandibular prognathism and genes, such as growth hormone receptor ( *GHR* ), 13 erythrocyte membrane protein band4.1 ( *EPB4* ), 14 synovial sarcoma X (SSX21), myosin1H ( *MYO1H* ), 15 collagen type-II α1 ( *COL2A1* ), 16 fibroblast growth factor ( *FGF7* ), 17 transforming growth factor beta 3 ( *TGFB3* ), plexin A ( *PLXNA* ), latent transforming beta binding protein 2 ( *LTBP2* ), matrilin1( *MATN1* ), 18 dual specificity phosphatase 6 ( *DUSP6* ), 19 a disintegrin and metalloproteinase with thrombospondin motifs 1 ( *ADAMTS1* ), 20 in various populations. SNPs in certain genes have been associated with the incidence of various malocclusions. Genetic susceptibility, to a particular trait due to SNP where changes occur with a single-base substitution and create a single-nucleotide difference in two strands of DNA, is most commonly reported in various studies. The frequency of minor alleles is usually studied for their association with the trait in question that would indicate causality or elevated risk of developing or protecting a particular trait. *MYO1H* is a class-I myosin. It is a different protein group than the myosin isoforms of muscle sarcomeres which are the basis of skeletal muscle fiber typing. 21 The composition ratio of muscle fibers is greatly influenced by genetic factors and rarely by environmental factors. A wide variation of myosin heavy chain isoforms exists in the masseter muscle of people with different mandibular plane angles. Type-I myosin heavy chain isoform was the most common isoform found in all subjects. 22

Myosins are molecular motors that, upon interaction with actin filaments, use adenosine triphosphate hydrolysis to generate mechanical force. Myosin-I generate movement at the leading edge in cell motility, phagocytosis, and vesicle transport. 23 Since myosins are involved in these biologic pathways, we could speculate that muscular functions play an essential role in mandibular growth. This gene *MYO1H* maps on chromosome 12, at 12q24.11 according to the Entrez Gene. Multiple lines of evidence have indicated that the orofacial musculature influences craniofacial morphology. 24 25 26 Genetic alterations that affect muscle would also affect the adjoining skeletal areas. This is consistent with the functional matrix hypothesis, in which skeletal growth is linked to its underlying muscular matrix. 27 Few studies conducted before reported that rs10850110 of *MYO1H* as a risk factor for mandibular prognathism in American, Malay, and Romanian populations. We performed SNP genotyping of rs10850110 of the *MYO1H* gene using the PCR-RFLP method to analyze the association of this polymorphism in the development of skeletal class-III malocclusion due to mandibular prognathism in our local population.

The results of our study were similar to that of a few of the studies conducted in other populations, which showed that the G allele being overrepresented when compared with the “A” allele in mandibular prognathism cases ( *p* < 0.05), and this was very significant. Also, in our study, the genotypes in the overdominant model showed an association with mandibular prognathism with an OR at a 95% CI of 1.0 with a *p* -value of 0.72. The frequency of the less common rs10850110 allele varies from 0.008 in sub-Saharan Africans to 0.089 in Japanese, 0.148 in Han Chinese, and 0.275 in Europeans. 28 The “A” allele variant may be associated with a CTCF binding site (intronic variant) and affect the muscular activity and mandibular growth functions. The frequency of the less common rs10850110 allele, that is, A allele, was higher in cases than controls in the present study than that reported from other populations.

Tassopoulou-Fishell et al 29 conducted a study wherein they used PCR along with TaqMan chemistry to amplify the genome and to trace polymorphisms. A correlation was found between the rs10850110 polymorphism of the *MYO1H* gene and mandibular prognathism in their study population. Though he analyzed the role of other polymorphisms rs2503243, rs972054, rs1413533, rs1490055, rs2101560, rs1601948, rs1387168, rs2940913, rs7718944, rs3016534, and rs9458378 in mandibular prognathism, they could not find any correlation. This shows that rs10850110 *MYO1H* is an important marker in analyzing mandibular prognathism. Sun et al 30 used the whole-mount in situ hybridization (WISH) technique to analyze the pattern of expression of *MYO1H* and they concluded that *MYO1H* plays a vital role in mandibular growth because of the involvement of this gene in the proliferation and morphology of the mandibular condyle chondrocytes.

Cruz et al 31 used the TaqMan method of real-time PCR to amplify the genome and demonstrated that the polymorphism of rs10850110 in *MYO1H, GHR* , and *FGF* genes plays a role in mandibular prognathism and maxillomandibular discrepancies. Yahya et al 32 on the Malay population also showed the role of SNP (rs10850110) *MYO1H* in class-III mandibular prognathism patients as a risk factor. Da Fontoura et al on Romanian and American populations showed that the G allele of rs10850110 polymorphism of the *MYO1H* gene being overrepresented in skeletal class-III patients. A marker (rs11066446) upstream of *MYO1H* was also reported to be associated with a principal component of skeletal malocclusion variations which explained the horizontal maxillomandibular discrepancies. 33 Dalaie et al 34 in the Iranian population and Cunha et al 35 in the Brazilian population conducted a similar study wherein they found no significant correlation between rs10850110 of *MYO1H* and skeletal class III due to mandibular prognathism. In a review article, the role of *MYO1H* in mandibular prognathism in different populations was reported recently. 36 Future studies with larger sample sizes are required on different races and ethnic groups. Also, other SNPs of this gene should be assessed in future studies to determine its association in the development of mandibular prognathism.

## Conclusion

The polymorphism rs10850110 in *MYO1H* , mapped on locus 12q24.11, is associated with an increased risk of skeletal class-III malocclusion due to mandibular prognathism. This study will lay the foundation for future studies to identify the exact role and mechanism of *MYO1H* polymorphism affecting mandibular growth. The identification of genetic influences in malocclusions helps in the prevention and improve treatment modalities of maxillomandibular discrepancies.
